# Lifetime and past-year prevalence of children’s exposure to violence in 9 Balkan countries: the BECAN study

**DOI:** 10.1186/s13034-017-0208-x

**Published:** 2018-01-02

**Authors:** George Nikolaidis, Kiki Petroulaki, Foteini Zarokosta, Antonia Tsirigoti, Altin Hazizaj, Enila Cenko, Jelena Brkic-Smigoc, Emir Vajzovic, Vaska Stancheva, Stefka Chincheva, Marina Ajdukovic, Miro Rajter, Marija Raleva, Liljana Trpcevska, Maria Roth, Imola Antal, Veronika Ispanovic, Natasha Hanak, Zeynep Olmezoglu-Sofuoglu, Ismail Umit-Bal, Donata Bianchi, Franziska Meinck, Kevin Browne

**Affiliations:** 1 Department of Mental Health and Social Welfare, Centre for the Study and Prevention of Child Abuse and Neglect, Institute of Child Health, 7 Fokidos Str., 11526 Athens, Greece; 2 Children’s Human Rights Centre of Albania, Tirana, Albania; 3 Faculty of Political Sciences, University of Sarajevo, Sarajevo, Bosnia and Herzegovina; 4 Department of Medical Social Sciences, South-West University “N. Rilski”, Blagoevgrad, Bulgaria; 5 Department of Social Work, Faculty of Law, University of Zagreb, Zagreb, Croatia; 6 University Clinic of Psychiatry, University of Skopje, Skopje, Former Yugoslav Republic of Macedonia; 7 Social Work Department, Faculty of Sociology and Social Work, Babes-Bolyai University, Cluj-Napoca, Romania; 8 Faculty for Special Education and Rehabilitation, University of Belgrade, Belgrade, Serbia; 9 Association of Emergency Ambulance Physicians, İzmir, Turkey; 10 Instituto degli Innocenti, Florence, Italy; 11 Centre for Evidence-Based Interventions, University of Oxford, Oxford, UK; 12 School of Behavioural Sciences, North-West University, Vanderbeijlpark, South Africa; 13 Centre for Forensic and Family Psychology (Division of Psychiatry and Applied Psychology), School of Medicine, University of Nottingham, Nottingham, UK; 14 Present Address: Department of Applied Mathematics and Computer Science, Technical University of Denmark, Copenhagen, Denmark; 15 Present Address: “The Smile of the Child”, Athens, Greece; 16 Present Address: Humanities and Social Sciences Department, University of New York Tirana, Tirana, Albania; 17 Present Address: AWO Clearinghaus for Unaccompanied Minor Refugees, Dortmund, North Rhine-Westphalia Germany

**Keywords:** Violence against children, Child abuse and neglect, Child maltreatment, Violence, Epidemiology, Balkans

## Abstract

**Background:**

Children’s exposure to violence is a major public health issue. The Balkan epidemiological study on Child Abuse and Neglect project aimed to collect internationally comparable data on violence exposures in childhood.

**Methods:**

A three stage stratified random sample of 42,194 school-attending children (response rate: 66.7%) in three grades (aged 11, 13 and 16 years) was drawn from schools in Albania, Bosnia and Herzegovina, Bulgaria, Croatia, Former Yugoslavian Republic of Macedonia (FYROM), Greece, Romania, Serbia and Turkey. Children completed the ICAST-C questionnaire, which measures children’s exposure to violence by any perpetrator.

**Results:**

Exposure rates for psychological violence were between 64.6% (FYROM) and 83.2% (Greece) for lifetime and 59.62% (Serbia) and 70.0% (Greece) for past-year prevalence. Physical violence exposure varied between 50.6% (FYROM) and 76.3% (Greece) for lifetime and 42.5% (FYROM) and 51.0% (Bosnia) for past-year prevalence. Sexual violence figures were highest for lifetime prevalence in Bosnia (18.6%) and lowest in FYROM (7.6%). Lifetime contact sexual violence was highest in Bosnia (9.8%) and lowest in Romania (3.6%). Past-year sexual violence and contact sexual violence prevalence was lowest in Romania (5.0 and 2.1%) and highest in Bosnia (13.6 and 7.7% respectively). Self-reported neglect was highest for both past-year and lifetime prevalence in Bosnia (48.0 and 20.3%) and lowest in Romania (22.6 and 16.7%). Experiences of positive parental practices were reported by most participating children in all countries.

**Conclusions:**

Where significant differences in violence exposure by sex were observed, males reported higher exposure to past-year and lifetime sexual violence and females higher exposure to neglect. Children in Balkan countries experience a high burden of violence victimization and national-level programming and child protection policy making is urgently needed to address this.

**Electronic supplementary material:**

The online version of this article (10.1186/s13034-017-0208-x) contains supplementary material, which is available to authorized users.

## Background

Violence against children has attracted gradually increasing clinical attention over recent decades. From its first reporting by the American pediatrician Henry Kempe in the 1960s [1] up to its recognition by the World Health Organization as a major public health issue in the late 1990s [2, 3], perspectives on the subject matter have changed drastically. During the last decades, violence against children has experienced increasingly interdisciplinary attention, first predominantly in social policy, social work, psychology and clinical practice and more recently also in public health. Reasons and causes of the phenomenon’s increased visibility over the years should be attributed to the literature on the severe implications of early exposure of children to violence or deprivation. Violence exposure in childhood is associated with negative physical and emotional health outcomes [4] which include anxiety and depression [5–7], suicidal ideation [8–10], substance use [11], dissociation and personality disorders, neurobiological implications [12] as well as with wider psychosocial consequences such as adolescent delinquency, educational shortcomings [13, 14], difficulties in relationships and family roles in adulthood, criminal activity [15] and reproduction of the “circle of violence” [16].

This paper follows the UNICEF definitions of violence against children and uses this interchangeably with the term children’s exposure to violence. Physical violence against children includes “all corporal punishment and all other forms of torture, cruel, inhuman or degrading treatment or punishment as well as physical bullying and hazing by adults or other children”. Psychological violence includes all “psychological maltreatment, mental abuse, verbal abuse and emotional abuse or neglect”. Sexual violence includes “any sexual activities imposed by an adult or child against which the child is entitled to protection by criminal law. […] Sexual activities are also considered as abuse when committed against a child by any other child if the offender is significantly older than the victim or uses power, threat or other means of pressure”. Neglect includes the “failure to meet children’s physical and psychological needs, protect them from danger or obtain medical, birth registration or other services when those responsible for their care have the means, knowledge and access to services to do so [17]”. Violence against children is thus more broadly defined than child abuse and neglect or child maltreatment.

Violence against children has over the past decade attracted international attention and its prevention and reduction has now been included into the Sustainable Development Goals [18]. There is currently a global interest to multiply efforts and join forces to eradicate children’s exposure to all forms of violence and increase awareness of the problem at global and local levels. An increasing number of countries across the globe have prohibited all forms of violence against children [19]. Of the nine countries participating in this study, Greece, Romania, Bulgaria and Croatia had enacted laws prohibiting violence against children in the home and school. Albania and Former Yugoslav Republic Of Macedonia (FYROM) joined them in 2010 and 2013, while Bosnia and Herzegovina, Serbia and Turkey have expressed commitment to law reforms banishing violence against children in all settings [19]. A recent systematic review found that attitudes condoning corporal punishment and other forms of violence against children decrease drastically in countries with legislation that bans all forms of violence against children, as do prevalence rates [20].

As a result, the necessity for building up a robust evidence base regarding the magnitude of the various types of children’s exposure to violence is becoming a necessity for the international scientific community in order to establish trends and changes in violence exposure over the years. One straightforward obstacle to this goal has traditionally been the radical incommensurability of results reported by various researchers around the globe using different tools and measuring fundamentally incompatible concepts of the phenomenon [21]. Moreover, it has been noticed that some of these tools measured subjective perceptions of exposure to violence and therefore suffered from decreased reliability [22].

To tackle such issues, during the last decade, the World Health Organization (WHO) and the International Society for the Prevention of Child Abuse and Neglect (ISPCAN) have initiated a set of recommendations for producing globally compatible and reliable data on measuring children’s exposure to violence [23]. This initiative was later supplemented by other similar organizations trying to specify optimum methodological requirements for conducting field research on violence against children [24]. The main characteristics of all such recommendations of international organizations [23, 25] involve applying credible and internationally used tools for inquiring about prevalence and incidence of children’s exposure to violence, using questionnaires measuring objective actions and experiences versus subjective perceptions of children’s victimization (i.e. asking “how many times have you been beaten, spanked, or smacked” instead of “have you experienced physical violence”). Further recommendations are to follow standardized methodologies of conducting research (e.g. using trained professionals instead of laymen as field researchers, designing strict protocols for research implementation to avoid biased suggestion of researchers’ attitudes and prejudices to participant subjects), and conducting field studies in representative randomly selected samples of the respective children’s general population in order for results to be a valid estimation of the actual situation in the referred population (in contrast with results deriving from clinical studies) [25].

On these grounds, with the support of the Oak Foundation, ISPCAN collaborated with UNICEF, the UN Secretary General’s Study on Violence against Children, the Office of the High Commissioner of Human Rights, and WHO to create the ISPCAN Child Abuse Screening Tools (ICAST) [26, 27] which allow the systematic collection and comparison of child abuse data concerning children’s exposure to violence by any perpetrator.

Within this overall framework the Balkan Epidemiological Child Abuse and Neglect (BECAN) project was undertaken and funded by EU’s 7th Framework Program for Research and Innovation (I.D.: 223478/HEALTH/2007) in order to establish past-year and lifetime prevalence of children’s exposure to violence in nine countries of the Balkan Peninsula. As there were no empirical data available on children’s exposure to violence up to the time of the particular research effort, the aim of this study was to investigate the epidemiology of violence against children in the participating countries for international comparisons and to serve as a baseline rate for future research.

## Methods

### Research design and sampling

**Fig. 1:**
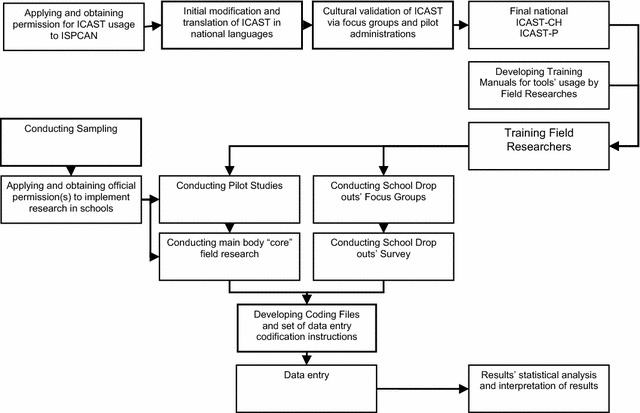
Field survey’s flowchart

The different steps in the research process are illustrated in Fig. 1.

The BECAN research project was a cross-sectional study of lifetime and past-year prevalence of children’s exposure to violence in the following nine countries: Albania, Bosnia and Herzegovina, Bulgaria, Croatia, Former Yugoslavian Republic of FYROM, Greece, Romania, Serbia and Turkey. The study utilized the ICAST-C questionnaire which was developed for use with children 11-years and older. This tool aims at measuring children’s self-reported exposure to various types of violence (by all potential perpetrators) and its items are structured in different sub-scales corresponding to children’s exposure to physical, psychological and sexual violence and neglect.

A three-stage stratified random sample was drawn from the general school-going population of 11, 13 and 16 year olds in the nine countries. First, official data about the child population and number of schools per region was obtained for the year preceding the study from the respective Offices of Statistics and the Ministries of Education in each country. These data constitute the sampling frame. Within the regions, schools were randomly selected into the sample using random series of numbers generated by a statistician until the number of schools was filled for each stratum. Since classes only partly equate age groups, students in grades reflecting the age clusters 11, 13 and 16 were recruited. All children who were part of that class, present on the day and consented, participated in the research. The vast majority of children in the participating countries attend school to age 18, therefore only school children were recruited for this present study.

The initial targeted sample was 63,250 children. This corresponds to 2–5% of the general population of children according to official figures released by the educational authorities of each country. The percentage varies with respect to the overall size of the population in each country, with smaller percentages in countries with larger populations. However, given the overall sample size and the randomized selection, the sample was regarded as representative of children attending schools in the participating countries.

### Measures

Physical, psychological and sexual violence exposure, neglect and positive and non-violent parenting were measured using the ICAST-C, a 38 item self-report measure for children developed by ISPCAN for prevalence studies across diverse contexts [26]. The ICAST measures past-year and lifetime prevalence of physical, psychological and sexual violence by any perpetrator, neglect and positive/non-violent parenting, similar to other instruments which have been used in prevalence studies in other European countries [28]. A limited amount of research is available on the validity and internal consistency of the ICAST-C. The measure showed good internal validity (Cronbrach’s alpha greater than 0.70) for the physical violence, psychological violence, sexual violence and neglect sub-scales across countries as diverse as China, Romania, Egypt, India, Russia, Columbia and Iceland in initial validation studies [26, 29, 30].

In accordance with ISPCAN’s rules and procedures, the ICAST-C was modified and subsequently translated into the official languages of the participating countries [31]. Modification was undertaken to align items with the parent version which is subject to a separate manuscript. Further, modifications were used to increase ease of reading and understanding by creating separate items for those questions which described multiple violent incidents. Translation was followed by cultural validation, back-translation and the development of a protocol for application of the measure. Small cultural modifications were made to describe specific practices in the different countries, i.e. frightening children with the bogeyman or by evoking evil spirits had to be translated into a locally relevant equivalent. The resulting measure was then subjected to a three round modification process including a consensus panel, 37 focus groups with 392 children and pilot studies in each of the countries (see Table 1 for number of focus groups conducted). These were conducted in rural and urban areas and recruited at last one classroom with pupils aged 11–16 (N = 1861). The focus groups aimed at elucidating whether children in all countries had the same cognitive and cultural understanding of the questions. The pilot studies collected 1331 modified ICAST-C questionnaires (response rate: 71.52%) and found that children in all age groups were able to understand and answer all items. The overall adaptation, piloting and consultation process across the nine countries took approximately 1 year.

**Table 1:** Number of focus groups that were conducted and number of children participating in them per country

Country	11 years olds		13 years olds		16 years olds		School dropouts	
	No of FGs	No of children	No of FGs	No of FGs	No of children	No of children	No of FGs	No of children
Albania	1	13	1	1	13	12	–	–
B&H	1	7	2	1	7	26	–	–
Bulgaria	1	14	1	1	14	11	1	6
Croatia	2	19	2	2	19	17	1	9
FYROM	1	16	1	1	16	17	1	4
Greece	1	8	1	1	2	7	–	–
Romania	–	–	2	2	18	36	1	9
Serbia	2	21	1	1	13	14	–	–
Turkey	1	8	1	1	9	7	–	–
Total	10	106	12	11	111	147	4	28

The final versions of the modified ICAST-C questionnaires comprised 45 items (children aged 11) and 51 items (adolescents aged > 12) structured in five scales. These measure exposure to psychological (17 items/19 items), physical (15 items/16 items), and sexual violence exposure (5 items/6 items), feelings of neglect (3 items) and reported experiences of nonviolent positive parental practices (5 items/7 items) which were added to the initial ICAST-C questionnaire [32]. For information on the actual phrasing of items please see Additional file 1. Each item inquired about specific violent events in the past year and allowed for the following response options: ‘once or twice a year’, ‘several times a year’, ‘monthly or every 2 months’, ‘several times a month’, ‘once a week or more often’, ‘not in the past year, but it has happened to me before’, ‘never in my life’ and ‘I don’t want to answer’. The final order of question items was informed by focus group discussions and expert opinion on the quality of children’s responses taking into account their age group and cognitive development [33]. The full questionnaire, as administered, can be viewed at http://becan.eu/sites/default/files/uploaded_images/EN_ICAST-CH.pdf.

*Socio*-*demographics* measured age of child, sex, whether child lives with mother, and urban/rural location of school.

### Research protocol

A standard protocol was developed for application of questionnaires to children in classrooms across the nine participating countries. Field researchers had to be certified professionals (psychologists and social workers). They received extensive training in interviewing vulnerable children about sensitive topics. Emphasis in training was placed on confidentiality, privacy and on neutrality during the interview process in order to avoid influencing children’s responses [34]. Questionnaires were self-administered in classrooms with interviewers present to answer questions or aid children if they got upset. Children with learning and physical disabilities were interviewed face-to-face. Children in the grade group aged 11 were asked the shorter 45 item version of the modified ICAST-C, children in the grade groups 13 and 16 were asked the longer 51 item version of the modified ICAST-C. Researchers in Turkey were unable to ask the questions about sexual abuse as government permission for this was not granted.

### Ethical issues

Permission to conduct the research in the school setting was granted by the educational authorities in each country. All children and their caregivers were informed in advance about the plans to carry out the research and provided consent. In line with in-country legislation, parental consent was either passive or active. However, a wide range of ethical and methodological issues emerged during the set-up of the field research relating to differences in national legislation and authoritative agency responses. These included, among others, the rights of disabled children to participate, the differentiation of oral versus written consent for parents and children and its implications or potential for parental refusal to participate in cases of severe child abuse. To deal with these issues, independent ethical advisory boards were set up in each country to provide supervision and guidance. These were overseen by an international independent ethics advisory board. Further, ad-hoc crisis intervention teams were set up in each country to help with collaborations between the research teams and local community agencies to facilitate referrals following child abuse disclosures where children were considered to be at risk of significant harm.

### Data entry and statistical analysis

Data were collected from all nine participating countries and entered into databases by trained professionals. Research teams double checked data entry and data quality on a regular basis. For past-year prevalence, items were dichotomized based on any vs no exposure in the past year on the different abuse sub-scales. For lifetime prevalence, items were dichotomized based on any vs no exposure in the past year or ever. This resulted in past-year prevalence rates for physical, emotional, sexual abuse, contact sexual violence exposure, neglect and positive parenting. Prevalence rates were then calculated using basic descriptive functions of the software package SPSS 18. Sex differences were assessed using *χ* ^*2*^ tests. Internal consistency of the different sub-scales of the ICAST-C measure were calculated using Cronbach’s alpha.

## Results

Participation rates differed between countries and school grades. Overall, 63,250 pupils were invited to participate in the survey. Of these 42,194 filled in a questionnaire resulting in a 66.7% response rate. Reasons for non-response included non-attendance at school on the day the survey was carried out, parental consent not obtained and child consent not obtained. Country-specific national participation rates ranged from 45.8% in FYROM to 82.7% in Turkey although a direct comparison is difficult between countries due to differences related to gaining parental consent (active–passive-none), enrolment numbers in school and actual student attendance throughout the school year. Participation rates by grade group and by country are presented in Table 2, in which the sample sizes are also presented. Socio-demographic characteristics of participants and their parents and location of school are described in Table 3.

**Table 2:** Description of schoolchildren’s sample and response rates by grade group and country

Country	Grade group									Total		
	11-year olds			13-year olds			16-year olds					
	N^1^	n^2^	R.R^3^	N^1^	n^2^	R.R^3^	N^1^	n^2^	R.R^3^	N^1^	n^2^	R.R^3^
Albania	1652	1186	71.79	1667	1204	72.23	1125	937	83.29	4444	3327	74.86
Bulgaria	1241	662	53.34	1105	685	61.99	1273	693	54.44	3619	2040	56.37
B & H	1333	676	50.71	1340	675	50.37	1501	1287	85.74	4174	2638	63.20
Croatia	1744	1223	70.13	1771	1188	67.08	1492	1233	82.64	5007	3644	72.78
Greece	4401	2771	62.96	5072	3438	67.78	5847	4242	72.55	15,320	10,451	68.22
FYROM	2058	670	32.56	2183	791	36.23	1408	1125	79.90	5649	2586	45.78
Romania	3471	1976	56.93	2709	1849	68.25	2190	2130	97.26	8370	5955	71.15
Serbia	2131	908	42.61	2623	1400	53.37	2811	1719	61.15	7565	4027	53.23
Turkey	2913	2500	85.82	3162	2564	81.09	3027	2462	81.33	9102	7526	82.69
Total	20,944	12,572	60.03	21,632	13,794	63.77	20,674	15,828	76.56	63,250	42,194	66.71

^1^N: number of children registered to schools that were included in the sample

^2^n: number of children who accepted to participate by filling in the ICAST-C questionnaire

^3^R.R.: response rate (percentage of the children who accepted to participate, out of the total number of invited school children in the selected school)

**Table 3:** Socio-demographic characteristics of the sample and location of schools

Country	School characteristics	Child characteristics			Parental characteristics
	In rural area	Age	Female	Lives with mother	Married
	% (n)	Mean (SD)	% (n)	% (n)	% (n)
Albania	46.0% (1530)	13.10 (2.05)	54.2% (1802)	96.5% (3212)	94.8% (3153)
Bulgaria	29.0% (592)	13.48 (2.04)	51.5% (1049)	88.8% (1812)	74.5% (1519)
B & H	36.5% (932)	14.26 (2.19)	53.1% (1400)	94.0% (2479)	86.5% (2282)
Croatia	27.5% (967)	13.59 (2.13)	51.1% (1863)	95.8% (3491)	84.9% (3094)
Greece	16.1% (1682)	13.78 (1.85)	52.4% (5480)	97.0% (10,137)	83.8% (8758)
FYROM	13.6% (226)	13.90 (2.17)	58.2% (967)	96.1% (1597)	87.7% (1458)
Romania	43.7% (2602)	13.73 (2.19)	55.5% (3305)	90.2% (5374)	81.0% (4825)
Serbia	35.8% (1441)	14.26 (2.12)	48.6% (1959)	94.9% (3821)	81.6% (3287)
Turkey	13.1% (983)	13.45 (2.14)	49.2% (3703)	93.6% (7046)	89.1% (6709)

### Internal consistency of the ICAST

Internal consistency of the various ICAST sub-scales was measured by calculating Cronbach’s alpha and is reported in Table 4. Internal consistency of the psychological violence sub-scale was good with Cronbach’s alpha ranging from 0.80 to 0.96. Internal consistency for physical violence was good to excellent with Cronbach’s alpha ranging from 0.81 to 0.99. Internal consistency of the sexual violence subscale was adequate to good with Cronbach’s alpha ranging from 0.71 to 0.86. Internal consistency of the contact sexual violence sub-scale was poor to adequate ranging from 0.41 to 0.76. Internal consistency of the neglect sub-scale was poor to good with Cronbach’s alpha ranging from 0.60 to 0.87. Internal consistency of the positive and non-violent parenting subscale was poor to good with Cronbach’s alpha ranging from 0.35 to 0.81.

**Table 4:** Internal consistencies (Cronbach’s alpha) of scales of exposure to psychological, physical and sexual violence, neglect and positive/non-violent parenting scales, by country

Country	Form of children’s exposure (scales of the ICAST-C^R.^)					
	Psychological violence	Physical violence	Sexual violence	Contact sexual violence	Feeling of neglect	Positive and non violent parenting
Albania	0.806	0.900	0.819	0.666	0.705	0.354
B & H	0.865	0.897	0.793	0.557	0.748	0.760
Bulgaria	0.816	0.796	0.705	0.411	0.753	0.672
Croatia	0.895	0.920	0.858	0.764	0.756	0.807
FYROM	0.827	0.852	0.772	0.624	0.712	0.705
Greece	0.830	0.892	0.828	0.645	0.601	0.723
Romania	0.833	0.887	0.840	0.715	0.734	0.672
Serbia	0.840	0.890	0.850	0.652	0.653	0.737
Turkey	0.963	0.992	N/A	N/A	0.873	0.732

*N/A* not available

### Lifetime and past-year prevalence rates of violence exposure by country

Aggregated results for lifetime and past-year prevalence are presented in Tables 5 and 6. Lifetime prevalence for physical violence ranged from 50.6% (FYROM) to 76.4% (Greece), while past year prevalence ranged from 42.5% (FYROM) to 51.0% (Bosnia). Lifetime prevalence for psychological violence ranged from 64.6% (FYROM) to 83.2% (Greece), while past-year prevalence ranged from 59.6% (Serbia) to 70.0% (Greece). Lifetime prevalence of sexual violence ranged from 7.9% (Romania) to 18.6% (Bosnia), while past-year prevalence ranged from 5.0% (Romania) to 14.6% (Bosnia). Lifetime prevalence of contact sexual violence ranged from 3.6% (Romania) to 9.8% (Bosnia), while past-year prevalence ranged from 2.1% (Bosnia) to 7.7% (Bosnia). Lifetime prevalence of feelings of neglect ranged from 22.6% (Romania) to 42.6% (Turkey), while past-year prevalence ranged from 16.7% (Romania) to 37.6% (Turkey). Lifetime prevalence of positive and non-violent parenting ranged from 83.9% (FYROM) to 98.2% (Greece), while past-year prevalence ranged from 83.0% (FYROM) to 96.2% (Greece).

**Table 5:** Lifetime prevalence of schoolchildren’s exposure to violent behaviors by form of violence experienced, by country

Country	Form of children’s exposure (scales of the ICAST-C^R.^)											
	Psychological violence		Physical violence		Sexual violence		Contact sexual violence		Feeling of neglect		Positive and non violent parenting	
	% (n)	95% C.I.	% (n)	95% C.I.	% (n)	95% C.I.	% (n)	95% C.I.	% (n)	95% C.I.	% (n)	95% C.I.
Albania	68.62 (2283)	67.04–70.20	59.44 (1977)	57.77–61.11	11.11 (369)	10.04–12.18	4.85 (161)	4.12–5.59	25.73 (854)	24.24–27.22	94.59 (3146)	93.82–95.36
B & H	72.51 (1912)	70.80–69.47	67.68 (1782)	65.89–69.47	18.68 (491)	17.19–20.17	9.75 (256)	8.61–10.88	39.63 (1042)	37.77–41.50	95.94 (2528)	95.19–96.69
Bulgaria	69.51 (1418)	67.51–71.51	62.21 (1269)	60.10–64.31	8.58 (175)	7.36–9.79	4.90 (100)	3.97–5.84	23.68 (483)	21.83–25.52	92.21 (1881)	91.04–93.37
Croatia	73.04 (2661)	71.60–74.49	66.73 (2425)	65.20–68.26	10.18 (369)	9.20–11.17	4.50 (163)	3.83–5.18	35.30 (1281)	33.74–36.85	97.23 (3539)	96.69–97.76
FYROM	64.58 (8691)	62.74–66.42	50.66 (7962)	48.73–52.59	7.60 (1645)	6.58–8.63	3.80 (787)	3.06–4.55	27.47 (3871)	25.74–29.19	83.87 (10,258)	82.45–85.29
Greece	83.16 (1670)	82.44–83.88	76.37 (1307)	75.56–77.19	15.86 (194)	15.16–16.57	7.60 (96)	7.08–8.11	37.20 (707)	36.27–38.13	98.18 (2168)	97.93–98.44
Romania	76.67 (4564)	75.59–77.74	66.94 (2974)	65.74–68.13	7.90 (467)	7.21–8.58	3.56 (210)	3.09–4.03	22.59 (1388)	21.52–23.65	95.97 (5710)	95.47–96.47
Serbia	68.44 (2756)	67.00–69.87	69.18 (2779)	67.75–70.61	8.49 (340)	7.62–9.35	4.90 (196)	4.23–5.57	28.83 (1157)	27.43–30.23	97.34 (3917)	96.84–97.84
Turkey	70.58 (5311)	69.55–71.61	58.38 (4384)	57.27–59.50	N/A^a^		N/A^a^		42.62 (3194)	41.50–43.73	93.91 (7060)	93.37–94.45

^a^Not available

**Table 6:** Past-year prevalence of schoolchildren’s exposure to violent behaviors by form of violence experienced, by country

Country	Form of children’s exposure (scales of the ICAST-C^R.^)											
	Psychological violence		Physical violence		Sexual violence		Contact sexual violence		Feeling of neglect		Positive and non violent parenting	
	% (n)	95% C.I.	% (n)	95% C.I.	% (n)	95% C.I.	% (n)	95% C.I.	% (n)	95% C.I.	% (n)	95% C.I.
Albania	61.71 (2053)	60.06–63.36	48.41 (1610)	46.71–50.10	9.12 (303)	8.14–10.10	4.07 (135)	3.40–4.74	21.84 (725)	20.44–23.25	92.96 (3092)	92.10–93.83
B & H	64.05 (1689)	62.22–65.88	51.01 (1343)	49.10–52.92	13.62 (358)	12.31–14.93	7.65 (201)	6.64–8.67	33.21 (873)	31.41–35.01	94.27 (2484)	93.38–95.16
Bulgaria	62.01 (1265)	59.90–64.12	48.48 (989)	46.31–50.65	7.50 (153)	6.36–8.64	4.36 (89)	3.48–5.25	19.90 (406)	18.17–21.63	90.15 (1839)	88.85–91.44
Croatia	65.69 (2393)	64.15–67.23	45.54 (1655)	43.92–47.16	7.20 (261)	6.36–8.04	3.26 (118)	2.68–3.84	28.63 (1039)	27.16–30.10	96.18 (3501)	95.56–96.80
FYROM	60.21 (7318)	58.32–62.10	42.40 (4939)	40.50–44.31	6.39 (989)	5.44–7.34	3.37 (461)	2.66–4.07	24.90 (2748)	23.23–26.57	83.02 (10,052)	81.57–84.46
Greece	70.02 (1557)	69.14–70.90	47.38 (1094)	46.42–48.33	9.54 (163)	8.97–10.10	4.45 (85)	4.05–4.85	26.41 (641)	25.56–27.25	96.21 (2146)	95.84–96.58
Romania	65.90 (3923)	64.70–67.10	44.65 (2651)	43.39–45.92	4.99 (295)	4.43–5.54	2.09 (123)	1.72–2.45	16.66 (987)	15.71–17.61	93.19 (5545)	92.55–93.83
Serbia	59.62 (2401)	58.11–61.14	46.48 (1867)	44.94–48.02	6.24 (250)	5.49–6.99	3.70 (148)	3.11–4.28	22.85 (917)	21.55–24.15	94.58 (3806)	93.88–95.28
Turkey	62.82 (4727)	61.73–63.91	46.06 (3459)	44.94–47.19	N/A^a^		N/A^a^		37.55 (2814)	36.45–38.64	90.74 (6822)	90.09–91.40

^a^Not available

### Lifetime differences in violence exposure by sex

Differences between males and females in relation to lifetime violence exposure were examined. No differences were observed in relation to lifetime psychological violence exposure between males and females across countries (see Table 7). For lifetime physical violence exposure, no differences could be observed between sexes across countries except for Turkey, where males reported higher prevalence of physical violence than females (60.6% vs 56.1%). For lifetime sexual violence exposure, no differences were observed between sexes amongst the majority of countries except for Albania, where males reported higher lifetime sexual violence exposure than females (14.5% vs 8.2%) and FYROM, where this was also the case (9.6% vs 6.0%). For lifetime contact sexual violence exposure, differences between males and females could be observed with higher lifetime prevalence among males in Albania (8.1% vs 2.1%), Bosnia (12.3% vs 7.7%), FYROM (5.5% vs 2.5%) and Serbia (6.0% vs 3.8%). For lifetime experiences of feelings of neglect, differences between males and females could be observed with higher lifetime prevalence among females in Albania (30.7% vs 19.8%), Bosnia (47.5% vs 30.8%), Croatia (40.6% vs 29.8%), FYROM (31.0% vs 23.1%), Greece (42.8% vs 31.0%), Romania (26.6% vs 17.6%), Serbia (34.6% vs 23.4%) and Turkey (48.1% vs 37.3%). No differences between sexes were observed for lifetime positive and non-violent parenting (Table 7).

**Table 7:** Lifetime-prevalence of schoolchildren’s exposure to violent behaviors by form of violence experienced and by child’s sex, per country

Country	Sex	Form of children’s exposure (scales of the ICAST-C^R.^)											
		Psychological violence		Physical violence		Sexual violence		Contact sexual violence		Feeling of neglect		Positive and non violent parenting	
		% (n)	95% C.I.	% (n)	95% C.I.	% (n)	95% C.I.	% (n)	95% C.I.	% (n)	95% C.I.	% (n)	95% C.I.
Albania	Female	70.09 (1263)	67.97–72.20	60.65 (1093)	58.40–62.91	8.22 (148)	6.95–9.49	2.06 (37)	1.40–2.72	30.74* (553)	28.61–32.87	95.23 (1716)	94.24–96.21
	Male	66.93 (1018)	64.57–69.29	58.16 (884)	55.68–60.64	14.50* (220)	12.73–16.27	8.11 (123)	6.74–9.49	19.79 (300)	17.78–21.79	93.95 (1428)	92.75–95.15
B & H	Female	73.36 (1027)	71.04–75.67	67.43 (944)	64.97–69.88	17.93 (251)	15.92–19.94	7.65 (107)	6.26–9.04	47.50* (665)	44.88–50.12	96.57 (1352)	95.62–97.52
	Male	71.67 (878)	69.15–74.20	68.25 (834)	65.64–70.86	19.47 (237)	17.25–21.70	12.25 (149)	10.41–14.10	30.79 (375)	28.20–33.38	95.42 (1167)	94.25–96.59
Bulgaria	Female	68.83 (722)	66.02–71.63	59.87 (628)	56.90–62.83	7.91 (83)	6.28–9.55	4.29 (45)	3.06–5.52	25.93 (272)	23.28–28.58	92.56 (971)	90.98–94.15
	Male	70.23 (696)	67.39–73.08	64.58 (641)	61.71–67.66	9.28 (92)	7.48–11.09	5.55 (55)	4.12–6.98	21.29 (211)	18.74–23.84	91.83 (910)	90.12–93.53
Croatia	Female	73.54 (1370)	71.53–75.54	66.38 (1236)	64.23–68.53	11.96 (222)	10.48–13.44	5.18 (96)	4.17–6.18	40.56* (754)	38.33–42.79	97.91 (1823)	97.26–98.56
	Male	72.53 (1291)	70.45–74.60	67.10 (1189)	64.91–69.29	8.31 (147)	7.03–9.60	3.80 (67)	2.90–4.69	29.77 (527)	27.64–31.90	96.51 (1726)	95.66–97.37
FYROM	Female	63.70 (4590)	61.21–66.18	49.03 (4236)	46.44–51.61	6.01 (907)	4.78–7.24	2.47 (423)	1.66–3.28	30.96* (2343)	28.57–33.36	83.66 (5394)	81.75–85.57
	Male	65.68 (4101)	62.93–68.43	52.71 (3726)	49.82–55.60	9.64* (738)	7.91–11.37	5.50 (364)	4.16–6.84	23.07 (1528)	20.62–25.52	84.13 (4864)	82.02–86.25
Greece	Female	83.76 (916)	82.78–84.74	77.37 (704)	76.26–78.48	16.62 (86)	15.63–17.61	7.76 (35)	7.05–8.47	42.83* (444)	41.52–44.14	98.43 (1203)	98.10–98.76
	Male	82.50 (754)	81.44–83.55	75.27 (603)	74.07–76.47	15.02 (108)	14.03–16.02	7.42 (61)	6.68–8.15	30.96 (263)	29.67–32.25	97.91 (965)	97.51–98.30
Romania	Female	76.91 (2542)	75.48–78.35	65.57 (2163)	63.94–67.19	7.90 (260)	6.98–8.82	3.01 (99)	2.43–3.60	26.56* (876)	25.05–28.07	96.43 (3187)	95.80–97.06
	Male	76.51 (2003)	74.88–78.18	68.79 (1794)	67.01–70.57	7.91 (205)	6.87–8.95	4.26 (110)	3.48–5.04	17.57 (456)	16.10–19.03	95.37 (2949)	94.57–96.18
Serbia	Female	71.31 (1397)	69.31–73.31	68.57 (1342)	66.52–70.63	7.53 (147)	6.36–8.70	3.79 (74)	2.95–4.64	34.56* (676)	32.45–36.67	97.96 (1919)	97.33–98.58
	Male	65.72 (1359)	63.67–67.76	69.76 (1437)	67.77–71.74	9.39 (193)	8.13–10.65	5.95 (122)	4.92–6.97	23.38 (481)	21.55–25.21	96.76 (1998)	95.99–97.52
Turkey	Female	70.89 (2625)	69.43–72.35	56.12 (2077)	54.52–57.72	N/A^a^		N/A^a^		48.12* (1780)	46.51–49.73	94.65 (3502)	93.92–95.37
	Male	70.28 (2686)	68.83–71.73	60.58* (2307)	59.03–62.14	N/A^a^		N/A^a^		37.25 (1414)	35.71–38.79	93.19 (3558)	92.39–93.99

^a^Not available

* Significant at p < 0.05

### Past-year differences in violence exposure by sex

Differences between males and females in relation to past-year violence exposure were examined. In relation to past-year prevalence, no significant differences were observed in relation to psychological violence exposure apart from in Serbia with females reporting higher exposure (63.3% vs 56.2%). For past-year prevalence of physical violence, differences between males and females were observed with higher levels of exposure for males in Romania (47.7% vs 42.3%) and Turkey (48.5% vs 43.6%). For past-year sexual violence, higher levels of exposure were observed for males in Albania (12.9% vs 6.0%), FYROM (8.3% vs 4.9%) and Serbia (7.5% vs 5.0%). For past-year contact sexual violence, higher levels of exposure were observed for males in Albania (7.3% vs 1.4%), Bosnia (10.0% vs 5.7%), FYROM (4.8% vs 2.3%), Greece (5.5% vs 3.5%), Romania (2.9% vs 1.5%) and Serbia (4.8% vs 2.5%). For past-year exposure to feelings of neglect, higher levels of exposure were observed for females in Albania (26.7 vs 16.1%), Bosnia (40.5% vs 25.0%), Croatia (33.7% vs 23.3%), FYROM (28.75 vs 20.1), Greece (30.9% vs 21.5%), Romania (19.4 vs 13.1%), Serbia (27.7% vs 18.3%) and Turkey (43.1% vs 32.1%). No differences between sexes were observed for past-year positive and non-violent parenting (Table 8).

**Table 8:** Past-year prevalence of schoolchildren’s exposure to violent behaviors by form of violence experienced and by child’s sex per country

Country	Sex	Form of children’s exposure (scales of the ICAST-C^R.^)											
		Psychological violence		Physical violence		Sexual violence		Contact sexual violence		Feeling of neglect		Positive and non violent parenting	
		% (n)	95% C.I.	% (n)	95% C.I.	% (n)	95% C.I.	% (n)	95% C.I.	% (n)	95% C.I.	% (n)	95% C.I.
Albania	Female	63.37 (1142)	61.15–65.60	48.83 (880)	46.53–51.14	6.00 (108)	4.90–7.10	1.39* (25)	0.85–1.93	26.68* (480)	24.64–28.73	93.40 (1683)	82.25–94.54
	Male	59.83 (901)	57.37–62.29	48.03 (730)	45.51–50.54	12.85* (195)	11.17–14.54	7.26 (110)	5.95–8.56	16.09 (244)	14.25–17.94	92.57 (1407)	91.25–93.88
B & H	Female	65.93 (923)	63.45–68.41	49.79 (697)	47.17–52.40	12.43 (174)	10.70–14.16	5.65* (79)	4.44–6.86	40.50* (567)	37.93–43.07	95.50 (1338)	94.41–96.59
	Male	62.04 (760)	59.32–64.76	52.62 (643)	49.82–55.42	15.04 (183)	13.03–17.05	10.03 (122)	8.34–11.72	25.04 (305)	22.61–27.47	93.13 (1139)	91.71–94.55
Bulgaria	Female	61.77 (648)	58.83–64.71	47.28 (496)	44.26–50.30	6.96 (73)	5.42–8.50	3.72 (39)	2.57–4.86	22.21 (233)	19.70–24.73	90.75 (952)	89.00–92.51
	Male	62.66 (621)	59.65–65.68	49.75 (493)	46.63–52.86	8.07 (80)	6.38–9.77	5.05 (50)	3.68–6.41	17.46 (173)	15.09–19.82	89.51 (887)	87.60–91.41
Croatia	Female	66.40 (1237)	64.25–68.54	44.58 (830)	42.32–46.83	8.03 (149)	6.79–9.26	3.34 (62)	2.52–4.16	33.67* (626)	31.53–35.82	96.78 (1802)	95.98–97.58
	Male	64.94 (1156)	62.73–67.16	46.56 (825)	44.24–48.88	6.33 (122)	5.20–7.47	3.17 (56)	2.36–3.99	23.33 (413)	21.36–25.30	95.56 (1699)	94.60–96.51
FYROM	Female	59.81 (3833)	57.27–62.34	40.18 (2550)	37.65–42.72	4.89* (484)	3.77–6.01	2.26* 191)	1.49–3.04	28.73* (1689)	26.39–31.07	82.82 (5293)	80.87–84.77
	Male	60.71 (3485)	57.89–63.54	45.19 (2389)	42.31–48.08	8.30 (505)	6.69–9.92	4.78 (270)	3.52–6.03	20.09 (1059)	17.76–22.41	83.26 (4759)	81.10–85.42
Greece	Female	69.95 (860)	68.73–71.16	46.58 (577)	45.25–47.90	8.87 (70)	8.11–9.62	3.50* (32)	3.01–3.99	30.88* (412)	29.65–32.10	96.59 (1191)	96.11–97.07
	Male	70.11 (697)	68.83–71.38	48.26 (517)	46.87–49.65	10.28 (93)	9.43–11.13	5.50 (53)	4.86–6.14	21.45 (229)	20.31–22.60	95.79 (955)	95.23–96.35
Romania	Female	66.02 (2182)	64.41–67.64	42.29* (1395)	40.60–43.97	4.65 (153)	3.93–5.37	1.46* (48)	1.05–1.87	19.44* (641)	18.09–20.79	93.59 (3093	92.75–94.42
	Male	65.93 (1726)	64.11–67.74	47.70 (1244)	45.78–49.62	5.40 (140)	4.53–6.27	2.86 (74)	2.22–3.51	13.10 (340)	11.80–14.39	92.77 (2426	91.78–93.76
Serbia	Female	63.25* (1239)	61.11–65.38	45.94 (899)	43.73–48.15	4.92* (96)	3.96–5.88	2.51* (49)	1.82–3.21	27.66* (541)	25.68–29.64	95.05 (1862	94.09–96.01
	Male	56.19 (1162)	54.05–58.33	46.99 (968)	44.84–49.15	7.49 (154)	6.36–8.63	4.82 (99)	3.90–5.75	18.28 (376)	16.61–19.95	94.14 (1944)	93.13–95.15
Turkey	Female	63.06 (2335)	61.50–64.61	43.61* (1614)	42.01–45.21	N/A^a^		N/A^a^		43.09* (1594)	41.50–44.69	91.73 (3394)	90.84–92.62
	Male	62.59 (2392)	61.05–64.12	48.45 (1845)	46.86–50.04	N/A^a^		N/A^a^		32.14 (122)	30.65–33.62	89.79 (2418)	88.82–90.75

^a^Not available

* Significant at p < 0.05

## Discussion

This paper provides data on psychological, physical and sexual violence exposure, feelings of neglect and positive parenting from the Balkan Epidemiological Study of Child Abuse and Neglect (BECAN). It is the first study to examine past-year and lifetime prevalence in multiple countries in the region and the first to use cross-country comparable methodology to do so. The BECAN study used the ICAST-C measure to investigate prevalence of violence exposure in nationally representative samples of 11, 13 and 16 year olds in nine Balkan countries. The ICAST-C is a non-proprietary child violence exposure screening tool that has been designed for use in international research on the prevalence of violence against children and showed good internal consistency in this sample.

Investigating the international epidemiology of children’s violence exposure is important, not only for developing monitoring systems in the participating countries, but also for sensitizing and mobilizing communities to engage in child protection efforts. The results presented in this study provide an insight to the magnitude of the phenomenon of children’s exposure to violence in countries with no prior quantitative research data [35–37]. Moreover, data presented here also provide a baseline measurement for future research and can be used for the evaluation of large-scale social policies on child protection. Overall, the findings of this research documented in quantitative terms a considerable rate of children’s exposure to various harmful practices in the participating countries.

### Psychological violence

Rates of exposure to psychological violence were found to be high with the vast majority of children reporting past-year and lifetime exposure. Children’s self-reported exposure to psychological violence ranged from 64.6 to 83.2% for lifetime and 58.3 to 70.0% for past-year exposure. As with other studies from the region, except for Serbia where girls reported higher levels of exposure to past-year psychological violence, no significant differences in exposure between males and females could be observed [38]. However, lifetime prevalence rates in this study far exceeded the estimated European prevalence 29.2%, established by a recent meta-analysis which included six European studies [38]. A recent study in Romania using the Adverse Childhood Experiences Questionnaire in 15-year old students found a lifetime prevalence of 39.7% for psychological violence which is higher than the European mean but lower than the 77% found by this present study [39]. Further research is needed to establish the underlying drivers of these high rates of psychological violence in the region.

### Physical violence

Rates of physical violence exposure were found to be high with almost every second child reporting past-year exposure and more than every second child reporting lifetime victimization. Equivalent percentages of children’s self-reports for exposure to physical violence range from 50.7 to 76.4% for lifetime and 42.4 to 51.0% for past-year victimization. As with other studies from the region, apart from in two countries, no significant differences in physical violence exposure between males and females could be observed [40]. However, lifetime prevalence rates for physical violence exposure in this study far exceeded the European estimate of 22.9% established by a recent meta-analysis which included 19 European studies [40]. A recent study in Romania found a lifetime prevalence of 32.2% for physical violence among 15-year olds which is considerably lower than the 67% found by this present study [39]. Further research is needed to establish the underlying drivers of these high rates of physical violence in the region.

### Sexual violence

Rates of sexual violence exposure were found to range from one in twelve to one in six children for lifetime exposure and between one in twenty and one in ten children for past-year prevalence. Equivalent percentages of children’s self-reported exposure to contact sexual violence ranged from 2.1 to 7.7% for the last year and 3.5 to 9.8% across the lifespan. While exposure to sexual violence is typically more often associated with female victimization [41] in this study self-reported experiences of boys were found to exceed or equal girls’ self-reported exposures. In particular, boys in Albania, Bosnia and Herzegovina, FYROM, Greece, Romania and Serbia reported higher levels of contact sexual violence exposure compared to girls. This is contrary to findings from a recent meta-analysis of 39 publications which established lifetime prevalence of childhood sexual victimization in Europe as 13.5% for females and 5.6% for males, therefore finding lower prevalence of sexual victimization in boys [42]. The global prevalence estimates of sexual abuse in childhood in this meta-analysis also established higher risk for sexual victimization among girls. Recent research from Saudi Arabia and South Africa finds equal exposures for sexual victimization between boys and girls [43, 44]. Why boys report equal or increased exposure to sexual violence than girls in some regions of the world is unclear. Further research, is required to investigate the reasons for these elevated rates of sexual abuse victimization among boys in the participating countries.

### Neglect

Rates of subjective feeling of neglect were found to range from one in four to one in two children for lifetime exposure and between one in six and one in three children for past-year prevalence. Equivalent percentages of children’s self-reports for neglect experiences range from 16.7 to 37.5% for the last year and 22.6 to 42.6% across the lifespan. Rates of feeling neglected were reported significantly more by female children across almost all countries. A recent meta-analysis of 16 studies on emotional neglect could not establish a prevalence rate for Europe as it could not find any studies from the region [45]. However, the overall lifetime global prevalence estimate for emotional neglect was 18.4% which is lower than the estimates in this study. Further this meta-analysis found no difference in lifetime prevalence between boys and girls. Why girls report equal or increased exposure to neglect than boys is unclear although it may be related to the way in which the questions were framed as they did not ask about specific incidents but a general feeling of being uncared for. Further research is required to investigate the reasons for these elevated rates of neglect among girls in the participating countries.

### Positive discipline

Over 90% of participants reported exposure to positive and non-violent parenting. This is in stark contrast to the high numbers of violence exposure also reported in this study. One possible explanation for this phenomenon could be that caregivers make use of a range of disciplinary methods which may include harsh and physical punishment but can also include positive discipline techniques. Another possible explanation is that violence was perpetrated by a range of people in the child’s network such as peers, teachers and other relatives rather than just by the caregivers. It is also possible that despite thorough piloting, the questions on positive discipline were not precise enough for participants to understand them correctly. It is likely, that a combination of all three occurred. Further research is required to investigate the performance of the positive and non-violent parenting sub-scale in this sample.

Overall, prevalence of past-year and lifetime violence exposure varied across countries while few statistically significant differences in violence exposure were detected between boys and girls. The most noteworthy difference is that in sexual violence exposure which was more commonly reported by boys.

This study found much higher prevalence rates across all measured violence exposures compared to statistics released by the World Health Organization in 2016. This may be due to differences in design and the use of a more comprehensive questionnaire for the measurement of children’s exposure to violence which covered multiple domains and a vast array of violent incidents. It may also be due to differences in participant’s ages with younger children generally more likely to be exposed to physical violence and neglect while older children are more likely to be exposed to psychological and sexual violence [46].

## Limitations

Since the current study is a large-scale, international, cross-sectional study some common limitations in interpreting results have to acknowledged. First, this study utilized a child self-report measure which may be prone to recall and social desirability bias of responders. However, self-report by children is more reliable than parental report or agency records [47] and research has shown a tendency to under-report abusive experiences in studies using retrospective recall rather than over-report these [48]. Further, care was taken to ensure privacy and confidentiality throughout the research phase to reduce social desirability bias. Second, minor differences in implementation of the research protocol occurred across the different country sites. However, utmost care was taken to follow the protocol as closely as possible and to deviate only out of legal or practical necessity. Third, response-rates showed large variations across countries but no data could be collected with regards to the non-responding students and there is therefore the potential that this study excludes children that are most vulnerable to violence exposure. Recruitment rates did not differ according to consent procedure used (active vs passive) and neither did disclosure rates of violence exposure. Fourth, although utmost care was taken with the translation of the ICAST-C, there may be slight variations in phrasing across the multiple countries and languages in this study. Sixth, this study only included children enrolled in schools and thus might exclude children who are very vulnerable and out of school. However, pilot studies in the participating countries found that the vast majority of children in the target age groups were enrolled in schools due to mandatory education requirements up to age 18. Seventh, since participating countries have different age distribution of their child population, the samples were drawn using different proportions of 11-, 13- and 16-year old children according to the proportion of this population in the respective country. This should be taken into account particularly when interpreting age aggregated prevalence rates and is one of the reasons why this study does not conduct analyses to compare prevalence rates of violence exposure across the various countries. However, it should also be noted that despite geographical proximity, participating countries have substantial differences in a number of characteristics which are expected to influence prevailing behaviors in societies. Furthermore, it should be also taken into account that some of the participating countries experienced war or civil unrest less than a decade prior to conducting the surveys. This can influence societies’ prevailing behaviors and perspectives which could have influenced results in a number of different ways (from actual differences in prevalence of violence against children to differences in responding to such a survey). Finally, this study did not adjust for multiple comparisons based on Rothman’s suggestion that this will lead to fewer errors of interpretation when the data under evaluation are actual observations [49].

## Conclusions

Research on children’s exposure to violence has an increased social utility function over and above providing epidemiological evidence which can help predict the burden of mental health. Providing a robust evidence base for the understanding of the phenomenon of children’s victimization can ultimately facilitate effective social and child protection policy design and implementation. From this angle, current evidence indicates new targets for social policies and awareness raising interventions that could tackle currently invisible aspects of the phenomenon of children’s exposure to violence. In this context, this particular study generated a first quantitative measurement of the magnitude of the problem in the participant countries and served as a tool for awareness raising among professional communities and policy makers. It created a space for further research not just to verify its findings, but also for shedding more light on all aspects of children’s victimization which include medical, mental, psycho-social and human rights challenges for modern societies.

## Additional file

**Additional file 1.** BECAN ICAST-CH Scales.
