# Supplementary material for: Lifetime and past-year prevalence of children’s exposure to violence in 9 Balkan countries: the BECAN study
Source: Child Adolesc Psychiatry Ment Health. 2018 Jan 2;12:1. doi: 10.1186/s13034-017-0208-x (PMC5749026; doi:10.1186/s13034-017-0208-x)
Supplement: Supplementary file 1 — Additional file 1. BECAN ICAST-CH Scales. [file 13034_2017_208_MOESM1_ESM.docx]

**BECAN ICAST-CH Scales**

**(numbers of items as mentioned in the BECAN ICAST-CH, also correspond to ICAST-CH numbering with modifiers such as letters or numbers, viz. “A” or “.1”)**

All items use the following response code:

Once or twice a year, several times a year, monthly or every two months, several times a month, once a week or more often, not in the past year but it has happened to me before, never in my life, I don’t want to answer

*Sometimes, when children or adolescents are growing up, people say or do things, some of which could make the child or adolescent feel embarrassed, ashamed or bad. In the past year, has anyone in your family and living in your home:*

Psychological Violence

Items*

18A. Shouted, yelled, or screamed at you very loud and aggressively?

19A. Insulted you by calling you dumb, lazy or other names like that?

19B. Cursed you?

19.1. Refused to speak to you (ignored you)?

**19.2. Blamed you for his/her bad mood?**

19.10. Read your diary, your SMS or e-mail messages without your permission?

19.11. Went through your bag, drawers, pockets etc. without your permission?

19.12. Compared you to other children in a way that you felt humiliated?

20A. Ashamed or embarrassed you intentionally in front of other people in a way that made you feel very bad or humiliated?

21. Said that they wished you were dead or had never been born?

22. Threatened to leave you or abandon you?

22.1. Threatened to kick you out of house or send you away?

23. Locked you out of the home?

**24A. Threatened to invoke ghosts or evil spirits, or harmful people against you?**

24B. Threatened to hurt or kill you?

26A. Did not get enough to eat (went hungry) and/or drink (were thirsty) even though there was enough for everyone, as a means of punishment?

27A. Have to wear clothes that were dirty, torn, or inappropriate for the season, as a means of punishment?

37A. Locked you up in a small place or in a dark room?

40. Threatened you with a knife or a gun?

*Sometimes, people can hurt children and adolescents physically. Thinking about yourself, in the past year, has anyone in your family done something such as:*

Physical Violence

Items*

32A. Pushed or kicked you?

32.1. Grabbed you by your clothes or some part of your body and shook you?

33A. Slapped you?

33B. Hit you on head with knuckle or back of the hand?

33C. Spanked you on the bottom with bare hand?

34A. Hit you on the buttocks with an object such as a stick, broom, cane, or belt?

34B. Hit you elsewhere (not buttocks) with an object such as a stick, broom, cane, or belt?

34.1. Hit you over and over again with object or fist (“beat-up”)?

35A. Choked you or smothered you (prevent breathing by use of a hand or pillow) or squeezed your neck with hands (or something else)?

36A. Intentionally burned or scalded you?

**36B. Put chilli pepper, hot pepper, or spicy food in your mouth (to cause pain)?**

37B. Tied you up or tied you to something using a rope or a chain?

38A. Roughly twisted your ear?

38B. Pulled your hair?

38C. Pinched you roughly?

39A. Forced you to hold a position that caused pain or humiliated you as a means of punishment?

*Sometimes people do sexual things or show sexual things to children and adolescents. Thinking about yourself, has anyone familiar to you or an unknown person ever made you feel bad or uncomfortable by doing any of these things to you?*

Sexual Violence

Items*

41. Made you upset by speaking to you in a sexual way or writing sexual things about you?

42. Made you watch a sex video or look at sexual pictures in a magazine or computer when you did not want to?

43. Made you look at their private parts or wanted to look at yours?

44. Touched your private parts in a sexual way, or made you touch theirs?

**45A. Made a sex video or took photographs of you alone, or with other people, doing sexual things?**

46. Tried to have sex with you when you did not want them to?

*Sometimes, when children are growing up, people who are responsible for caring fo r them do not know how to care for children properly, and the children do not get what they need to grow up healthy. Have any of these things happened to you in the past year?*

Neglect

Items

28. Not taken care of when you were sick or injured - for example not taken to see a doctor when you were hurt or not given the medicines you needed?

29. You did not feel cared for?

30. Felt that you were not important?

31. Felt that there was never anyone looking after you, supporting you, helping you when you most needed it?

Positive Parenting

Items*

**19.3. Told you to start or stop doing something (e.g. start doing your homework or stop watching TV)?**

19.4. Explained you why something you did was wrong?

19.5. Gave you an award for behaving well?

**19.6. Gave you something else to do in order to distract your attention (e.g. to tell you do something in order to stop you watching TV)?**

19.7. Took away your pocket money or other privileges?

19.8. Forbade you something that you liked?

19.9. Forbade you to go out?

* Items in bold had been excluded from the short-version of the ICAST-CH completed by the 11 y-o grade’s pupils
